# Cyclic Glycine-Proline Improves Memory and Reduces Amyloid Plaque Load in APP/PS1 Transgenic Mouse Model of Alzheimer's Disease

**DOI:** 10.1155/2023/1753791

**Published:** 2023-03-01

**Authors:** Tushar Arora, Shiv K. Sharma

**Affiliations:** National Brain Research Centre, Manesar, 122052 Haryana, India

## Abstract

Alzheimer's disease (AD) is a neurodegenerative condition that is pathologically characterized by the presence of amyloid plaques and neurofibrillary tangles. Animal models of AD have been useful in understanding the disease process and in investigating the effects of compounds on pathology and behavior. APP/PS1 mice develop amyloid plaques and show memory impairment. Cyclic glycine-proline (cGP) is a cyclic dipeptide that is likely produced from a tripeptide, glycine-proline-glutamate, which itself is generated after proteolytic cleavage of insulin-like growth factor-1. Here, we show that cGP improves spatial memory and reduces amyloid plaque burden in APP/PS1 mice. The results thus suggest that cGP could potentially provide beneficial effects in AD.

## 1. Introduction

Alzheimer's disease (AD) accounts for majority of the dementia cases in the elderly population. Accumulation of misfolded proteins is a key neuropathological feature of AD. Intracellularly, neurofibrillary tangles containing hyperphosphorylated tau and, extracellularly, amyloid plaques are found in AD brains. These deposits cause cellular dysfunction and eventually cell death. The widely accepted hypothesis of AD, the amyloid cascade hypothesis [1], posits amyloid-*β* (A*β*) as the central player in the development of pathology in this disease. The A*β* peptide is produced by the proteolytic processing of amyloid precursor protein (APP) [2], and it exists in different forms including the oligomeric form. Studies have shown that the oligomeric A*β* causes oxidative stress, neuroinflammation, and toxicity to neurons [3–6]. Importantly, oligomeric A*β* causes synaptic dysfunction and deficits in synaptic plasticity and memory [3, 7–9].

Transgenic animals are widely used to model AD and study its pathology and behavioral deficits. The double transgenic mice with mutations in APP and presenilin-1 (PS1), commonly known as APP/PS1 mice, are widely used animal model for AD. These mice develop amyloid plaques and show impairment in memory [10].

Insulin-like growth factor-1 (IGF-1) plays crucial roles during brain development [11]. Intranasal administration of IGF-1 is a noninvasive method to deliver it to the brain [12]. This route of administration bypasses the blood-brain barrier. It has been shown that intranasally delivered IGF-1 provides protection against focal cerebral ischemia-induced damage [13]. An association between decreased level of IGF-1 and higher risk of developing AD has been reported suggesting that increasing the level of this growth factor may provide protection against neurodegeneration [14]. Alteration in IGF-1 signaling pathway has also been reported in AD [15, 16]. Further, IGF-1 reduces amyloid *β* burden [17].

IGF-1 is processed into an N-terminal tripeptide, glycine-proline-glutamate (GPE), and des-N-(1-3)-IGF-1 [18, 19]. Cyclic glycine-proline (cGP), likely a derivative of GPE, is another important metabolite of IGF-1 [19]. The cyclic structure confers this dipeptide resistance to enzymatic breakdown and greater lipophilicity for enhanced uptake to the brain [20]. cGP has been shown to be a regulator of IGF-1 homeostasis by modulating the binding of IGF-1 to its binding proteins [21]. However, the effects of cGP in AD are not known. In this study, we examined the effects of cGP on spatial memory and amyloid plaque load in APP/PS1 double transgenic mouse model of AD.

## 2. Materials and Methods

### 2.1. Animals

The double transgenic mice, B6;C3-Tg(APPswe,PSEN1dE9)85Dbo/Mmjax (APP/PS1), originally obtained from The Jackson Laboratory, were bred in the animal facility of National Brain Research Centre. These mice overexpress a chimeric mouse/human APP (Mo/HuAPP695swe) and a mutant human presenilin-1 (PS1-dE9) under a prion promoter.

Mice from the colony were tested for the presence of the transgenes using the APP and the PS1 primers [22]. Nine- to eleven-month-old male mice weighing 20-40 g, containing the transgenes (APP/PS1), and wild-type (WT) mice without the transgenes were used for the experiments. Mice were kept under 12 h light/12 h dark cycle in the animal facility, and the experiments were conducted during the day phase. Standard rodent chow and water were provided to the mice *ad libitum*. The procedures were approved by the Institutional Animal Ethics Committee.

### 2.2. Treatment with cGP

The animals were randomly distributed into the control and the experimental groups to avoid any bias. Each animal was handled for 5-10 min for 5 days. cGP (Cyclo(-Gly-Pro); Bachem, cat. no. G-1720) was dissolved in phosphate-buffered saline (PBS; 137 mM NaCl, 2.7 mM KCl, 10.14 mM Na_2_HPO_4_, 1.76 mM KH_2_PO_4_, and pH 7.4) at a concentration of 80 *μ*g/*μ*l and administered intranasally at a dose of 20 mg/kg body weight. Taking clues from previous studies that used a dipeptide analogue of piracetam [23] or graphene quantum dot-conjugated GPE [24], cGP treatment was done for 28 days. The control groups received the same volume of PBS as the volume of cGP. The dose of cGP was determined based on a pilot experiment (Supplementary Figure 1).

### 2.3. Spatial Memory Task

We used the Morris water maze (MWM) task, which has been extensively employed in the literature, to study spatial memory. It has been shown that the APP/PS1 mice show deficit in spatial memory [24, 25].

#### 2.3.1. Apparatus

The task was carried out in a circular pool, 1.68 m in diameter and 60 cm in height. The pool was half-filled with water, and the water temperature was kept at 24 ± 2°C. A circular platform (10 cm in diameter, made of white plexiglass) was placed 1 cm below the water surface. To hide the platform, a nontoxic white paint was mixed in water to make it opaque. At a distance of 40-60 cm around the pool, four objects (a tall multishelf rack, a large toy, a rectangular white paper sheet with a black cross, and a black cardboard box) were put as spatial cues. One-hundred-watt electric bulbs facing the roof were placed at the four corners of the room to keep the pool illuminated. The positions of the platform in the pool and the spatial cues around the pool were kept constant throughout the training phase. The platform was removed during the testing period while the spatial cues were retained at their original places.

#### 2.3.2. Training

WT mice and APP/PS1 mice were trained in MWM task using a modified version of the paradigm used previously [26]. The training was carried out for 8 days overlapping with last 8 days of drug treatment. During the training, each animal was given a total of 4 trials per day with an intertrial interval of 10 min. The first trial for each animal started at least 1 h after it received the drug. The maximum time gap between the drug administration and the first trial was 3 h.

Once gently released, the animals were allowed to freely explore the pool for 2 min and find the hidden platform. If they failed to find the platform within the stipulated time, they were manually directed to the platform and placed on it. The mice stayed on the platform for 30 seconds. The release positions were pseudorandomized each day. Escape latency was calculated as the time taken by the animals to reach the platform after their release in the water. The escape latency values in the 4 trials each day were averaged to get a single value for each animal on that day.

#### 2.3.3. Testing

The testing (probe trial) was conducted 24 h after the last training session to assess memory. In probe trial, each animal was given 2 min to explore the pool. The spatial memory was assessed using the parameters, escape latency (time taken to reach the former platform area), number of times the animals crossed the annulus (former platform area), and time spent in the platform quadrant (the quadrant which housed the platform during the training).

To record the behavior of the animals during training and testing phases, a webcam (Logitech) was installed above the pool. The path followed by the animals in the pool was tracked using ANYMAZE tracking software (ANYMAZE). The videos were collected in a hard disk and analyzed manually.

### 2.4. Thioflavin-S Staining

A separate set of mice was used for these experiments. WT and APP/PS1 mice received cGP intranasally for 28 days. They were sacrificed 4-5 h after receiving the drug on the 28^th^ day. The animals were anaesthetized with halothane and decapitated. The brains were taken out and washed with ice-cold PBS. The two hemispheres of the brain were separated. The left hemisphere of the brain from half of the animals and the right hemisphere of the brain from other half of the animals were taken and fixed overnight in 4% paraformaldehyde. In the next day, the samples were transferred to a 20% sucrose solution in PBS until they sank to the bottom, after which they were transferred to a 30% sucrose solution in PBS until they sank to the bottom.

The samples were sectioned into 40 *μ*m thick sections using cryostat (Leica CM3050 S) and placed in the wells of 24-well cell culture plates containing PBS with 0.01% sodium azide. Every 6^th^ section of a hemisphere was mounted on a slide, with a total of 5 sections per hemisphere. A single slide contained mounted sections from all the experimental groups.

Thioflavin-S (Sigma-Aldrich; cat. no. T1892) is a benzothiazole dye that displays enhanced fluorescence on binding to fibrillar *β*-sheets. The sections were stained with thioflavin-S to visualize amyloid plaques [10]. The stained sections were imaged using a Leica DFC 320 camera connected to a Leica DM RXA2 microscope (excitation at 390 nm and emission at 428 nm). All the imaging parameters were set initially with the first section, and they were kept constant for all the later sections across all the groups. The plaque load was quantified using ImageJ (NIH). Regions of interest were defined using free-hand tool, along the contours of the hippocampus and cortex. The RGB images were converted to binary images (8 bits). Thresholding values were manually adjusted to identify the plaques. To make sure that the specified value incorporates all the plaques, the thresholded image was compared with the original RGB image. Quantification of the plaques was done using “Analyze Particles” plugin of ImageJ, and the following four parameters were evaluated: plaque density (plaque count per mm^2^), percentage area covered by plaques, size of remaining plaques, and total area defined for analysis. Raw data values from all the five sections of a particular hemisphere were averaged to get a single value for each animal. Only in the representative images shown in the figures, for clarity, the brightness and contrast values were adjusted identically in both groups.

### 2.5. Data Analysis

**Figure 1:**
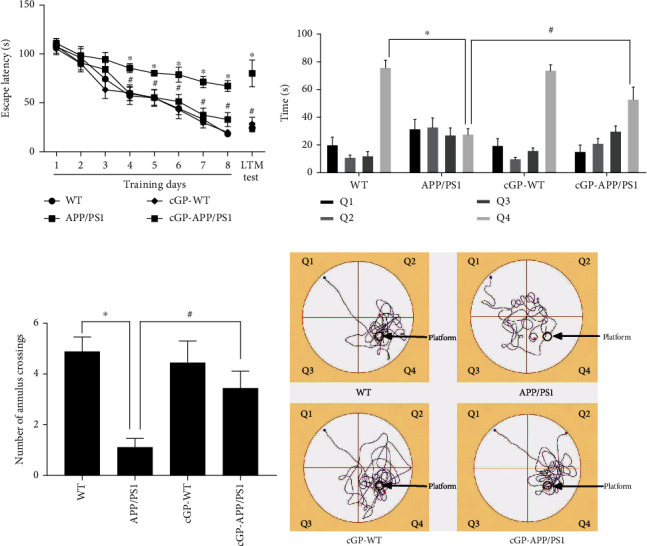
cGP improves memory in APP/PS1 mice. cGP treatment was given to wild-type (WT) and APP/PS1 mice. (a) Escape latency during the 8 days of training and during the long-term memory (LTM) test (*n* = 9 in all groups). There were significant differences in escape latency between APP/PS1 mice and WT mice day 4 onwards during the course of training and also during the LTM test. cGP-treated APP/PS1 (cGP-APP/PS1) mice showed significantly reduced escape latency compared to APP/PS1 mice day 4 onwards during the training and during the LTM test. Escape latencies of WT mice and cGP-treated wild-type (cGP-WT) mice were similar throughout the training and during the LTM test. (b) Time spent in different quadrants during the probe trial (LTM test in (a)). APP/PS1 mice spent significantly less amount of time in platform quadrant (Q4) than the WT mice. cGP-APP/PS1 mice spent significantly more time in the platform quadrant compared to untreated APP/PS1 mice. cGP-WT mice and WT mice spent similar amount of time in the platform quadrant. (c) Number of annulus crossings during the probe trial (LTM test in (a)). APP/PS1 mice displayed significantly reduced number of annulus crossings compared to WT mice. cGP-APP/PS1 mice showed significantly increased number of annulus crossings compared to untreated APP/PS1 mice. WT mice and cGP-WT mice showed similar number of annulus crossings. (d) The representative track plots during the probe trial (LTM test in (a)). Asterisks denote significant difference (*p* < 0.05) between the WT and APP/PS1 groups, and # denotes significant difference between the APP/PS1 and cGP-APP/PS1 groups.

**Figure 2:**
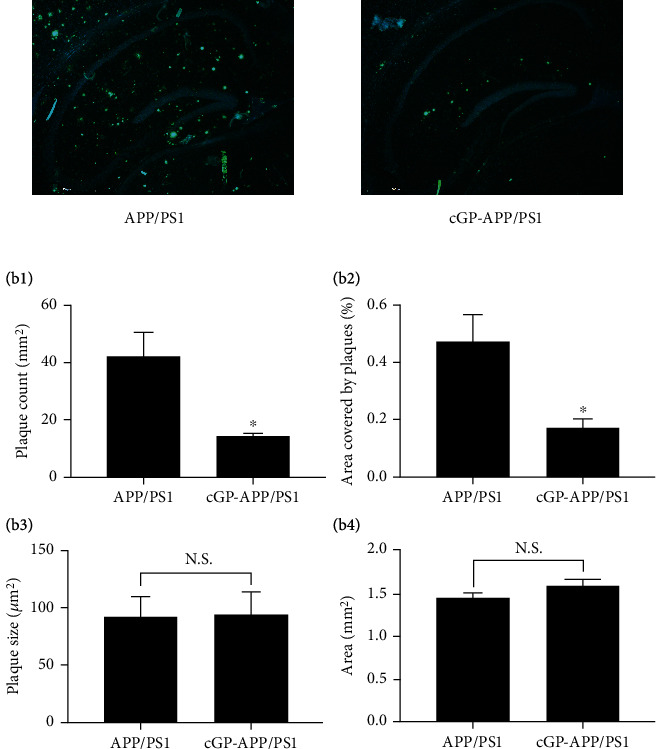
cGP treatment reduces amyloid plaque load in the hippocampus of APP/PS1 mice. Amyloid plaque load was evaluated in the hippocampus of APP/PS1 mice and cGP-treated APP/PS1 (cGP-APP/PS1) mice. Representative thioflavin-S-stained hippocampal sections from both groups of mice are shown in (a) (scale bar, 500 *μ*m). Treatment with cGP reduced the plaque density (b1) and percentage area covered by plaques (b2) in cGP-APP/PS1 mice (*n* = 4 in both groups). The average size of remaining plaques in cGP-APP/PS1 mice was similar to plaque size in APP/PS1 mice (b3), and total area used for plaque load analysis (b4) was similar in the two groups of mice. Asterisks denote significant difference (*p* < 0.05) compared to APP/PS1 group. N.S.: not significant.

**Figure 3:**
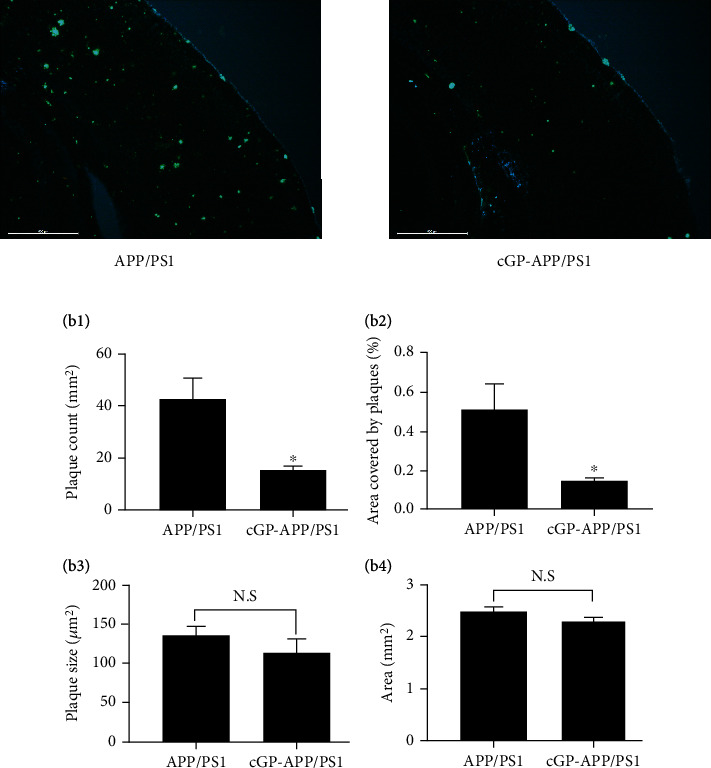
cGP reduces amyloid plaque load in the cortex of APP/PS1 mice. Amyloid plaque load was evaluated in the cortex of APP/PS1 and cGP-treated APP/PS1 (cGP-APP/PS1) mice. Representative thioflavin-S-stained cortical sections from both groups of mice are shown in (a) (scale bar, 500 *μ*m). cGP reduced plaque density (b1) and percentage area covered by plaques (b2) in APP/PS1 mice (*n* = 4 in both groups). The average size of remaining plaques in cGP-APP/PS1 mice was comparable to plaque size in APP/PS1 mice (b3). The total area used for plaque load analysis (b4) was similar in the two groups of mice. Asterisks denote significant difference (*p* < 0.05) compared to APP/PS1 group. N.S.: not significant.

The data presented in Figure 1 and supplementary Figures 2 and 3 are from one set of animals, and the data presented in Figures 2 and 3 are from another set of animals. Data in Figure 1 and supplementary Figures 2 and 3 were analyzed using two-way analysis of variance checking the main column effect by multiple comparisons. This was then followed by Fisher's least significant difference test where each comparison stands alone. In Figures 2 and 3, data were analyzed using unpaired Student's *t*-test (two-tailed). The group differences were considered significant when *p* value was < 0.05. Data are presented as mean ± SEM.

## 3. Results

### 3.1. cGP Treatment Improves Memory in APP/PS1 Mice

Several studies have shown that APP/PS1 mice are impaired in spatial memory [10, 25]. We asked whether cGP has any effect on spatial memory in APP/PS1 mice. For this purpose, we used the Morris water maze task, a widely used task to study spatial memory. cGP treatment was given to WT mice and APP/PS1 mice. Spatial learning and memory were examined during the 8 days of training and during the probe trial.  Figure 1(a) shows escape latency in the four groups of mice during the course of training and during LTM test. During training, significant difference in latency was found between WT and APP/PS1 mice showing that APP/PS1 mice are deficient in acquisition of the task (Figure 1(a), last day of training, escape latency in seconds, WT, 18.2 ± 2.8; APP/PS1, 67.2 ± 5.5, *p* < 0.01). Importantly, this deficit in acquisition was alleviated by treatment with cGP, as there was a significant difference in latency between untreated APP/PS1 mice and cGP-treated APP/PS1 mice (last day of training, escape latency in seconds, APP/PS1, 67.2 ± 5.5; cGP-APP/PS1, 32.9 ± 7.2, *p* < 0.01). The cGP-treated WT mice did not differ from WT mice indicating comparable learning in these two groups of mice (last day of training, escape latency in seconds, WT, 18.2 ± 2.8; cGP-WT, 19.9 ± 2.5, *p* > 0.8).

During the LTM test, the three parameters used to assess spatial memory were escape latency, time spent in the platform quadrant, and number of annulus crossings. Significant differences in all the three parameters were found between WT and APP/PS1 mice showing that APP/PS1 mice were deficient in LTM (Figure 1(a), LTM test, escape latency in seconds, WT, 24.4 ± 5; APP/PS1, 80 ± 13.8, *p* < 0.01; Figure 1(b), time spent in platform quadrant in seconds, WT, 76.1 ± 5.7; APP/PS1, 27.9 ± 4.4, *p* < 0.01; Figure 1(c), number of annulus crossings, WT, 4.9 ± 0.6; APP/PS1, 1.1 ± 0.4, *p* < 0.01). Importantly, the deficit in LTM observed in APP/PS1 mice was alleviated by treatment with cGP. There were significant differences in the three parameters between untreated APP/PS1 mice and cGP-treated APP/PS1 mice (Figure 1(a), LTM test, escape latency in seconds, APP/PS1, 80 ± 13.8; cGP-APP/PS1, 22.8 ± 3, *p* < 0.01; Figure 1(b), time spent in platform quadrant in seconds, APP/PS1, 27.9 ± 4.4; cGP-APP/PS1, 53.2 ± 9.2, *p* < 0.01; Figure 1(c), number of annulus crossings, APP/PS1, 1.1 ± 0.4; cGP-APP/PS1, 3.4 ± 0.7, *p* < 0.02). cGP-treated WT mice did not differ in these parameters from WT mice indicating comparable LTM in these two groups of mice (Figure 1(a), LTM test, escape latency in seconds, WT, 24.4 ± 5; cGP-WT, 28.3 ± 7, *p* > 0.7; Figure 1(b), time spent in platform quadrant in seconds, WT, 76.1 ± 5.7; cGP-WT, 74 ± 4.4, *p* > 0.8; Figure 1(c), number of annulus crossings, WT, 4.9 ± 0.6; cGP-WT, 4.4 ± 0.9, *p* > 0.6).  Figure 1(d) shows representative track plots depicting the path taken by the WT, APP/PS1, cGP-WT, and cGP-APP/PS1 groups of mice during the probe trial. Thus, consistent with previous findings, the results show that APP/PS1 mice are deficient in spatial memory. Importantly, cGP improves memory in these mice.

Swimming speed was assessed during the course of training and during the probe trial in the WT, APP/PS1, cGP-WT, and cGP-APP/PS1 groups of mice. All groups of mice showed similar swimming speeds across training and testing phases. There were no significant differences in swimming speeds of APP/PS1, cGP-WT, and cGP-APP/PS1 mice when compared to WT mice during training. During the probe trial also, the swimming speeds of APP/PS1, cGP-WT, and cGP-APP/PS1 mice did not differ from WT mice (Supplementary Figure 2).

Furthermore, weight of mice from all the four groups was measured before and after treatment with cGP. The mice in different groups had similar body weight before the start of cGP treatment. Further, cGP treatment did not affect the body weight of the mice (Supplementary Figure 3).

### 3.2. cGP Reduces Amyloid Plaque Load in the Hippocampus and Cortex of APP/PS1 Mice

The results in the previous section show that treatment of APP/PS1 mice with cGP alleviates deficit in spatial memory. Previous studies have shown amyloid plaque deposition in the brains of APP/PS1 mice. Thus, we assessed the effects of cGP on amyloid pathology in the hippocampus and cortex of APP/PS1 mice. cGP treatment was given to WT mice and APP/PS1 mice, and amyloid plaque load was assessed in brain sections after staining with thioflavin-S.

In the hippocampus, no amyloid plaques were observed in WT mice and cGP-WT mice. The representative images presented in Figure 2(a) show that APP/PS1 mice displayed a substantial number of amyloid plaques and cGP reduced the number of plaques in the hippocampus of these mice. There was a significant reduction in plaque density and percentage area covered by plaques in cGP-treated APP/PS1 mice compared to untreated APP/PS1 mice (Figure 2(b)b1, plaque count/mm^2^, APP/PS1, 42.3 ± 6.9; cGP-APP/PS1, 14.5 ± 0.6; *p* < 0.01; Figure 2(b)b2, percentage area covered by plaques, APP/PS1, 0.5 ± 0.1; cGP-APP/PS1, 0.2 ± 0.03; *p* < 0.03). The average size of remaining plaques in cGP-treated APP/PS1 mice was similar to plaque size in untreated APP/PS1 mice (Figure 2(b)b3, plaque size in *μ*m, APP/PS1, 92.8 ± 17.6; cGP-APP/PS1, 94.7 ± 19.9; *p* > 0.9). The total area used for analysis was comparable between APP/PS1 mice and cGP-APP/PS1 mice (Figure 2(b)b4, area in mm^2^, APP/PS1, 1.5 ± 0.1; cGP-APP/PS1, 1.6 ± 0.1; *p* > 0.2). The results presented here show that cGP reduces amyloid plaque load in the hippocampus of APP/PS1 mice.

The effect of cGP on amyloid pathology in the cortex of the same mice was also assessed. Similar to the hippocampus, no amyloid plaques were observed in WT mice and cGP-WT mice in the cortex. The representative images in Figure 3(a) show that a substantial number of amyloid plaques were present in the cortex of APP/PS1 mice and cGP reduced the number of plaques. There was a significant reduction in plaque density and percentage area covered by plaques in the cortex of cGP-treated APP/PS1 mice compared to untreated APP/PS1 mice (Figure 3(b)b1, plaque count/mm^2^, APP/PS1, 42.7 ± 7.7; cGP-APP/PS1, 15.4 ± 1.2, *p* < 0.02; Figure 3(b)b2, percentage area covered by plaques, APP/PS1, 0.5 ± 0.1; cGP-APP/PS1, 0.2 ± 0.01, *p* < 0.04). The average size of remaining plaques in the cGP-treated APP/PS1 mice was comparable to plaque size in the untreated APP/PS1 mice (Figure 3(b)b3, plaque size in *μ*m, APP/PS1, 136.2 ± 11.1; cGP-APP/PS1, 114.1 ± 17.1, *p* > 0.3. The total area analyzed in the cortex for plaque load was comparable between the APP/PS1 and cGP-APP/PS1 groups of mice (Figure 3(b)b4, area in mm^2^, APP/PS1, 2.5 ± 0.1; cGP-APP/PS1, 2.4 ± 0.1, *p* > 0.3). The results are similar to those observed in the hippocampus after cGP treatment of APP/PS1 mice. Collectively, the results show that cGP reduces amyloid pathology in two brain regions, i.e., the hippocampus and cortex of APP/PS1 mice.

## 4. Discussion

Alzheimer's disease is the most common cause of dementia in the geriatric population. Transgenic animals have been widely used to study the development of AD. These animal models show impairments in long-term potentiation and long-term depression [27]. Mice overexpressing mutated human APP show amyloid deposition and impairment in learning and memory [28, 29]. Double transgenic mice with mutations in both APP and PS1 show an accelerated rate of A*β* deposition in the brain [30]. Although the APP/PS1 mice develop amyloid plaques and show impairment in memory [24, 25], they do not develop AD-associated tau abnormalities. Thus, this model does not fully recapitulate human AD. However, the presence of amyloid plaques and cognitive decline make it suitable model for studying AD pathogenesis and for preclinical testing of potential therapeutic compounds. We used these mice to examine the effects of a cyclic dipeptide on spatial memory and amyloid plaque load in the hippocampus and cortex.

IGF-1 is essential for development [31]. In addition, this growth factor confers neuroprotection [32]. GPE and des-IGF-1 are the two fragments generated after proteolytic processing of IGF-1 [18, 19]. The effects of nanomaterial-conjugated GPE in animal model of AD have been examined earlier [24]. cGP is a metabolite of IGF-1 likely produced from GPE [19]. This cyclic dipeptide may possess enhanced lipophilicity for central penetration and may be resistant to proteolysis [20]. Therefore, compared to its parent peptide, cGP would be a preferred candidate to target the brain.

cGP and its analogue (NNZ 2591) have been shown to be neuroprotective in hypoxic-ischemic injury with NNZ 2591 even improving sensory-motor function [33]. Further, the same analogue inhibits memory impairment induced by scopolamine, likely by modulating acetylcholine neurotransmission [34], and protects against motor deficit in an animal model of Parkinson's disease [35]. However, the effects of cGP in AD have not been investigated. Therefore, we studied the effects of cGP on spatial memory and plaque load in APP/PS1 mouse model of AD. We find that this dipeptide ameliorates memory impairment as well as amyloid pathology in these mice.

The mechanisms involved in the effects of cGP on memory and amyloid plaque load are not known. cGP regulates IGF-1 homeostasis possibly by regulating its binding to the binding protein. cGP promotes IGF-1 activity when it is insufficient and inhibits IGF-1 activity when it is in excess [21]. Although the role of IGF-1 in AD is not clearly understood, studies have shown beneficial effects of this growth factor. For example, IGF-1 confers protection against A*β*- and mutant APP-induced toxicity [36, 37]. Increasing the level of IGF-1 reduces amyloid burden [17], and IGF-1 improves memory and reduces A*β* load in APP/PS2 mice [38]. Considering that cGP regulates IGF-1 activity, it is possible that the effects of cGP observed in this study on memory and amyloid plaque load involve, at least in part, IGF-1 signaling.

We have not determined the presence of cGP in the brain after intranasal administration. Previous studies have shown the presence of subcutaneously injected NNZ 2591 or intraperitoneally injected GPE in the cerebrospinal fluid [33, 39]. Thus, it is possible that intranasally administered cGP reaches the brain. Further studies are needed to determine the presence of cGP in the brain and to understand the molecular mechanisms underlying the effects of this peptide in AD transgenic mice.

## 5. Conclusions

A small cyclic peptide, cGP, endogenously generated from IGF-1, is shown to improve memory in APP/PS1 mice. This peptide reduces plaque load in the hippocampus and cortex of APP/PS1 mice. Hence, this could prove beneficial in mitigating AD pathology.

## Data Availability

The datasets generated during and/or analyzed during the current study are available from the corresponding author on reasonable request.

## Supplementary Materials

Supplementary Materials Supplementary information contains three figures and can be found within this article. Supplementary Figure 1: efficacy of different doses of cGP on escape latency in the Morris water maze task in APP/PS1 mice. Supplementary Figure 2: cGP treatment does not affect swimming speed of mice. Supplementary Figure 3: cGP treatment does not affect the weight of mice.
