# Supplementary material for: Cyclic Glycine-Proline Improves Memory and Reduces Amyloid Plaque Load in APP/PS1 Transgenic Mouse Model of Alzheimer's Disease
Source: Int J Alzheimers Dis. 2023 Mar 1;2023:1753791. doi: 10.1155/2023/1753791 (PMC9995210; doi:10.1155/2023/1753791)
Supplement: Supplementary Materials — Supplementary information contains three figures and can be found within this article. Supplementary Figure 1: efficacy of different doses of cGP on escape latency in the Morris water maze task in APP/PS1 mice. Supplementary Figure 2: cGP treatment does not affect swimming speed of mice. Supplementary Figure 3: cGP treatment does not affect the weight of mice. [file 1753791.f1.zip › Arora and Sharma Supplementary Material (Revised).doc]

**SUPPLEMENTARY MATERIAL**

**Supplementary Figure 1: Efficacy of different doses of cGP on escape latency in Morris water maze task in APP/PS1 mice.** cGP was administered to APP/PS1 mice in three different doses (5 mg/kg, 10 mg/kg and 20 mg/kg body weight) for 28 days (n = 2). They were subjected to spatial memory task during the last 8 days of cGP administration. APP/PS1 mice treated with 20 mg/kg body weight showed better performance compared to other groups of mice. Control, untreated APP/PS1 mice.

**Supplementary Figure 2**: **cGP treatment does not affect swimming speed of mice.** The swimming speed for each group of mice used in Figure 1 (main text) is shown. All the four groups of mice showed similar swimming speeds during the course of 8-day training and during the long-term memory (LTM) test. WT, wild type mice; cGP-WT, cGP treated wild type mice; APP/PS1, APP/PS1 mice; cGP-APP/PS1, cGP treated APP/PS1 mice.

**Supplementary Figure 3: cGP treatment does not affect the weight of mice.** The body weight of mice used in Figure 1 (main text) is shown. cGP did not affect the weight of animals. In case of “Post-cGP treatment,” the body weight of WT and APP/PS1 mice are the weights on the same day as the cGP treated mice (last day of cGP treatment) WT, wild type mice; cGP-WT, cGP treated wild type mice; APP/PS1, APP/PS1 mice; cGP-APP/PS1, cGP treated APP/PS1 mice.
